# Update on the campylobacter epidemic from chicken meat in New Zealand: The urgent need for an upgraded regulatory response

**DOI:** 10.1017/S095026882000299X

**Published:** 2020-12-15

**Authors:** M. G. Baker, L. Grout, N. Wilson

**Affiliations:** Department of Public Health, University of Otago, Wellington, New Zealand

**Keywords:** Campylobacter, epidemiology, food-borne infections, poultry, surveillance

## Abstract

New Zealand has a long-running campylobacter infection (campylobacteriosis) epidemic with contaminated fresh chicken meat as the major source. This is both the highest impact zoonosis and the largest food safety problem in the country. Adding to this burden is the recent rapid emergence of antibiotic resistance in these campylobacter infections acquired from locally-produced chicken. Campylobacteriosis rates halved in 2008, as compared with the previous 5 years, following the introduction of regulatory limits on allowable contamination levels in fresh chicken meat, with large health and economic benefits resulting. In the following decade, disease rates do not appear to have declined further. The cumulative impact would equate to an estimated 539 000 cases, 5480 hospitalisations, 284 deaths and economic costs of approximately US$380 million during the last 10 years (2009–2018). Additional regulatory interventions, that build on previously successful regulations in this country, are urgently needed to control the source of this epidemic.

## Text

In New Zealand, contaminated chicken meat is the largest single source of human campylobacteriosis. A huge increase in campylobacteriosis from 1980 to 2005 was closely correlated with the rise in fresh chicken meat consumption [1]. Rates of notifications and hospitalisations halved during 2007 after regulatory measures were introduced to reduce contamination levels in fresh chicken meat [2]. Key regulatory interventions introduced in 2005–2008 to reduce poultry-associated campylobacteriosis in New Zealand included monitoring and reporting the prevalence of *Campylobacter* spp. in caecal samples taken from birds from each growing shed each time birds were sent for processing; monitoring and reporting enumerated levels of *Campylobacter* spp. from rinsates of bird carcasses at the end of primary processing; mandatory targets for *Campylobacter* spp. contamination levels on poultry carcasses after primary processing; and intermittent monitoring of *Campylobacter* spp. contamination of retail poultry [2]. There was also a significant decline in Guillain-Barré syndrome (GBS), an uncommon but serious consequence of this infection [3]. However, even after a halving of notification rates after 2007, incidence remains high by global standards at 142 per 100 000 in 2018 [4]. This rate is more than seven times that of the USA (19.5 per 100 000 in 2018 [5]).

**Fig. 1.**
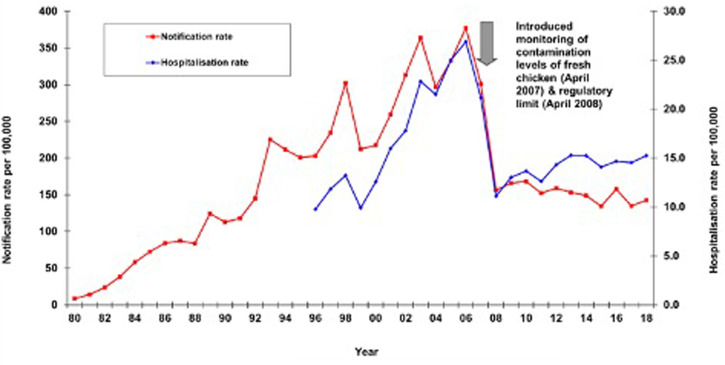
Campylobacteriosis notification and hospitalisation rates (cases per 100 000 per year), based on notification data from The Institute of Environmental Science and Research Limited (ESR) and hospital discharge data (principal and additional diagnosis) from the Ministry of Health.

Figure 1 illustrates the long-term rates of notification and hospitalisations. In the 11 years following the successful intervention in 2006–2007, the notification rate declined significantly (*P* < 0.012) by 8.5%, whereas hospitalisations for severe disease rose significantly (*P* < 0.005) by 38.7%. This pattern is of concern and suggests no substantive decline in campylobacteriosis in the last decade and potentially that the epidemic is increasing based on the large rise in hospitalisation rates.

Many epidemiological studies in New Zealand have implicated chicken meat in this epidemic [6, 7]. A recent high-quality case-control study with source attribution found that 84% of cases were infected with strains attributed to a poultry source of campylobacteriosis [8].

This ongoing foodborne epidemic has major health and economic consequences for New Zealand. Contaminated fresh chicken meat is estimated to cause more than 53900 symptomatic cases of human campylobacteriosis a year in this country. This estimate is based on the average number of notified cases of 6898 per year (2009–2018); a multiplier of 9.3 for estimating the number of community cases for each notified case, which was obtained from the Infectious Intestinal Diseases Study conducted in the UK [9]; and source attribution of 84% from poultry [8].

Campylobacteriosis is frequently a serious illness. It is by far the main cause of hospitalisation for a notifiable disease in New Zealand, with an average of 652 cases hospitalised each year (2009–2018). It is reasonable to estimate that contaminated chicken is responsible for around 84% of those hospitalisations (i.e., 548 per year). Around 30 of these infections will typically cause paralysis (i.e., GBS [3]) and others will result in serious invasive illness and death. The experience of an outbreak in the New Zealand town of Havelock North in August 2016 provides an idea of the potential mortality impact. This waterborne outbreak resulted in 7570 cases with four deaths, suggesting a mortality risk of approximately 52 per 100 000 cases [10]. Applied to the estimate of 539 000 chicken-associated cases, this suggests 284 deaths during the last 10 years or an average of 28 per year (2.4 per month) could be attributed to this particular food source. While this estimate is only a starting point for further research, it gives an indication of the potential scale of deaths from this contaminated food.

Contaminated chicken meat also provides a highly effective vehicle for disseminating antimicrobial resistance (AMR). A tetracycline and fluoroquinolone-resistant strain of campylobacter, first detected in chicken in 2014, spread rapidly across the North Island of New Zealand. By 2015 this strain was causing about a third of human campylobacteriosis cases in the country's largest city: Auckland [11]. The larger concern is that this episode shows how vulnerable the heavily contaminated poultry flocks in this country are to the entry and dissemination of AMR organisms.

Campylobacteriosis is the costliest foodborne disease in New Zealand, with estimated annual economic costs of NZ$134 million (m) in 2006–2007 (95% CI $101 to 172 m; with the point estimate equivalent to $170 m in 2019) [12]. Given a halving in incidence after 2007 and assuming 84% attribution to chicken, we can assign a minimum cost of $56 m (US$38 m) per annum to this specific contaminated food source. This cost is largely paid for by consumers suffering illness, employers and the government-funded health sector rather than by the poultry industry, which is the primary source.

As an island nation which produces almost all of its own poultry, New Zealand is well placed to take immediate action to better manage this epidemic. Responsible agencies, notably the Ministry of Primary Industries (MPI) and Food Standards Australia and New Zealand (FSANZ), should assign a high priority to control the campylobacteriosis epidemic linked to contaminated fresh chicken meat based on its huge human health and economic impact. MPI should regulate to markedly lower campylobacter levels permitted on fresh poultry. This intervention has proven success in reducing human infections from this source in the New Zealand setting [2]. Additionally, FSANZ should regulate to require high-quality consumer information labelling of poultry. There are currently minimal requirements for food safety labelling of fresh chicken meat sold in New Zealand [13], which is in stark contrast to large pictorial warning labels on tobacco products. If necessary, consumers should be actively shifted to safer food sources, beginning with restricting sales to only pre-cooked and frozen chicken products [14].

New Zealand's experience with this epidemic provides several important lessons that have relevance to the control of other foodborne diseases and public health problems more generally. First, slowly evolving epidemics can get less attention than they deserve. In general, a common source foodborne outbreak affecting more than a few dozen people typically results in an investigation and strong response if a source is identified. For example, the Havelock North outbreak of campylobacteriosis from contaminated water triggered a major inquiry [15, 16]. However, the country's well-characterised ‘common source’ campylobacteriosis epidemic is not receiving a vigorous response despite causing more human cases than all of New Zealand's reported foodborne disease outbreaks combined [17].

Effective public health surveillance of diseases and hazards is essential to guide improved food safety. However, overwhelming laboratory, epidemiological and economic evidence is not always enough to drive effective regulatory responses. Despite the growing quantity of high-quality research evidence about the role of contaminated chicken meat in causing the campylobacteriosis epidemic in New Zealand, there have been minimal further interventions in over a decade (from 2007 to 2019) and rates of notified and hospitalised disease do not suggest a decline in campylobacteriosis burden in that time.

Regulating food producers is more effective than educating consumers and is highly cost-effective [18]. It is difficult for chicken meat consumers to prevent infection from this highly contaminated food and cross-contamination from fresh chicken to other foods is a challenging problem and requires consistently good food handling practices by everyone involved in the food distribution chain. The scale and difficulty of this behaviour change are large compared with the proven benefits of reducing contamination levels on chicken supplied by producers [2].

Food safety interventions also depend on having a highly effective food safety regulator with an overriding focus on protecting public health and demonstrated independence from commercial interests. Ultimately, food safety regulation is a political process and benefits from advocacy from consumer groups and independent researchers who can provide informed critique of food safety issues. Regulators may be too cautious, under-resourced and constrained to act and may need external support to maintain momentum with enhancing food safety.

By every measure of importance, New Zealand's ongoing campylobacteriosis epidemic from contaminated chicken meat justifies a vigorous, multi-agency response. Adding up the impact of this epidemic during the last 10 years of inaction would equate to at least 539 000 cases of campylobacteriosis, 5480 hospitalisations, 284 deaths and economic costs to New Zealand of NZ$560 m (US$380 m).

The large Havelock North waterborne outbreak of campylobacter infection resulted in an exhaustive inquiry and a complete national reorganisation of the drinking-water supply sector [15, 16]. That common source outbreak caused about 7570 cases. By comparison, the ‘common source’ epidemic caused by contaminated chicken meat results in this number of campylobacteriosis cases every 2 months in New Zealand. It is probably time for a national inquiry to identify an effective response to this national epidemic from chicken meat. Responsibility for managing food safety also needs to shift to an independent regulator, potentially as part of a revitalised public health agency.

## Data Availability

The authors confirm that the data supporting the findings of this study are available within the article and its references.
